# Polyhydroxyalkanoate Production by Methanotrophs: Recent Updates and Perspectives

**DOI:** 10.3390/polym16182570

**Published:** 2024-09-11

**Authors:** Sanjay K. S. Patel, Deepshikha Singh, Diksha Pant, Rahul K. Gupta, Siddhardha Busi, Rahul V. Singh, Jung-Kul Lee

**Affiliations:** 1 Department of Chemical Engineering, Konkuk University, 120 Neungdong-ro, Gwangjin-gu, Seoul 05029, Republic of Korea; sanjaykspatel@hnbgu.ac.in (S.K.S.P.); guptarahul9m@gmail.com (R.K.G.);; 2 Department of Biotechnology, Hemvati Nandan Bahuguna Garhwal University (A Central University), Srinagar 246174, Uttarakhand, Indiapantdiksha8@gmail.com (D.P.); 3 Department of Microbiology, Pondicherry University, Pondicherry 605014, Kalapet, India; siddhardha.busi@gmail.com

**Keywords:** polyhydroxyalkanoates, methanotrophs, greenhouse gases, methane, biotransformation, environmental pollution

## Abstract

Methanotrophs are bacteria that consume methane (CH_4_) as their sole carbon and energy source. These microorganisms play a crucial role in the carbon cycle by metabolizing CH_4_ (the greenhouse gas), into cellular biomass and carbon dioxide (CO_2_). Polyhydroxyalkanoates (PHAs) are biopolymers produced by various microorganisms, including methanotrophs. PHA production using methanotrophs is a promising strategy to address growing concerns regarding plastic pollution and the need for sustainable, biodegradable materials. Various factors, including nutrient availability, environmental conditions, and metabolic engineering strategies, influence methanotrophic production. Nutrient limitations, particularly those of nitrogen or phosphorus, enhance PHA production by methanotrophs. Metabolic engineering approaches, such as the overexpression of key enzymes involved in PHA biosynthesis or the disruption of competing pathways, can also enhance PHA yields by methanotrophs. Overall, PHA production by methanotrophs represents a sustainable and versatile approach for developing biomedical materials with numerous potential applications. Additionally, alternative feedstocks, such as industrial waste streams or byproducts can be explored to improve the economic feasibility of PHA production. This review briefly describes the potential of methanotrophs to produce PHAs, with recent updates and perspectives.

## 1. Introduction

Synthetic plastics are an indispensable component of daily life, and a primary environmental concern is associated with their non-biodegradable nature. As the global energy demand continues to grow, the search for alternative and sustainable sources of energy has become increasingly important, for example, the conversion of biowaste into biogas, such as methane (CH_4_) by anaerobic digestion (AD) [1,2]. One promising avenue is the bioconversion of CH_4_, which is the primary component of natural gas, along with carbon dioxide (CO_2_), into liquid fuels and other valuable biochemicals [3,4,5]. Advances in bioreactor design and metabolic pathways engineering could allow microbes, such as methanotrophs, to convert CH_4_ into a variety of products, including polyhydroxyalkanoates (PHAs), fatty acids, single-cell proteins, exopolysaccharides (EPS), ectoine, putrescine, and α-humulene [6,7,8]. The natural gas reserve of CH_4_ is approximately 1.13 × 10^14^ m^3^ with high content (>95%), and global CH_4_ emissions are approximately 770 Tg/y. Methane (CH_4_) exhibits a global warming potential approximately 25-fold higher than CO_2_ [9,10,11]. The bioconversion of CH_4_ into liquid fuels and other valuable products, such as methanotrophs, has several advantages over traditional chemical conversion methods [12,13]. Biological conversion processes can operate at milder temperatures and pressures, potentially lowering the energy consumption and capital costs. Additionally, using CH_4_ for biofuel production can help mitigate greenhouse gas (GHGs) emissions by capturing and converting potent GHGs into sustainable fuel sources [6,10,14]. However, the large-scale implementation of CH_4_ bioconversion technology faces several challenges. Achieving high product yields and selectivity and addressing the inherent difficulties in gas–liquid mass transfer are critical hurdles that must be overcome [15,16,17]. Ongoing research and development in areas such as bioreactor design, enzyme engineering, and process optimization is crucial for realizing the full potential of methanotroph-based bioconversion of CH_4_ [18].

Polyhydroxyalkanoates (PHAs) are polymers with hydroxyalkanoic acid monomer units that can accumulate in a range of microbes, including heterotrophic bacteria and photoautotrophic organisms, such as cyanobacteria [19,20,21]. Methanotrophs are a unique group of microbes that use CH_4_ as their leading energy and carbon source [1,22]. These microbes flourish under harsh environmental conditions, such as low temperature, high hydrostatic pressure, and inadequate nutrients [23,24]. Methanotrophs utilize diverse substrates, such as CH_4_, formaldehyde, methanol, and methylamine, to produce numerous value-added biomolecules, including PHAs [3,6]. This property has drawn considerable attention from the scientific community because of its potential applications in bioremediation, biofuel production, and GHG mitigation. Methanotrophs such as *Methylosinus*, *Methylocystis*, *Methylocapsa*, *Methylomicrobium Methylococcus*, *Methylobacterium*, and *Methylocaldum* can accumulate these biopolymers (PHAs) as intracellular carbon (C) and energy storage compounds, amounting to more than 80% of cell dry weight (cdw) when grown under a nutrient-limiting environment, i.e., nitrogen (N) or phosphorus (P) limitation, with an excess of C source [1,6]. The AD process is a well-established technology for the conversion of organic waste into biogas (CH_4_ and CO_2_) [2]. The production of PHAs from inexpensive C sources, such as biogas-derived CH_4_, is vital for reducing costs. Acetogens, autotrophs, and methanotrophs can synthesize PHA using CH_4_, carbon monoxide, and CO_2_ [6,25]. Methanotrophs produce vital biomolecules for industrial and biotechnological applications [23,24]. The incorporation of unsaturated fatty acids into a ruminant’s diet can reduce CH_4_ production, which can indirectly affect the availability of CH_4_ for PHA production by methanotrophs. The notable challenges in converting CH_4_ into PHAs by methanotrophs include less efficient PHA-producing methanotrophs, low gas-to-liquid mass transfer efficiency, and inadequate properties of the synthesized PHAs, especially polyhydroxybutyrate (P(3HB)) [6,23,26]. Various strategies can be beneficial for achieving high PHAs using methanotrophs, including engineering microbes, high-molecular-weight (Mw) PHAs, the use of a gas-permeable membrane, two-phase partitioning bioreactors, co-polymers such as poly(3-hydroxybutyrate-co-hydroxyvalerate) (P(3HB-co-3HV)), and fast famine regimes [1,27]. Therefore, CH_4_ serves as a unique platform for the production of various chemicals using engineered methanotrophs [28,29]. In addition, methanotrophic bacteria are helpful in wastewater treatment systems, with the potential to produce biomolecules such as PHAs, ectoine, and methanobactins for broad biomedical applications, including drug carriers, electronic devices, heart stents, prosthetics, and vaccines [30,31]. The techno-economics of large-scale biorefinery (100,000 t/y) PHA production from CH_4_ by methanotroph analysis suggested that the production cost of PHAs varied between USD 4.1 and 6.8/kg assuming 25% of uncertainty [7]. Ongoing research efforts have focused on engineering methanotrophs to enhance their CH_4_ conversion capabilities further and to improve bioreactor design and operational strategies to optimize the overall process efficiency [1,5,6]. Scientists have developed robust and sustainable bioconversion systems that can transform CH_4_ into valuable chemicals and fuels by harnessing the unique metabolic capabilities of methanotrophs. Recent reviews have focused on the diverse aspects of methanotrophs to produce diverse types of bioproducts, with minor details and updates on PHA production [1,3,5,8,11,13]. In this review, we provide a brief update on the production of PHAs by methanotrophs from a future perspective.

## 2. Methanotrophs and Their Metabolism

**Figure 1:**
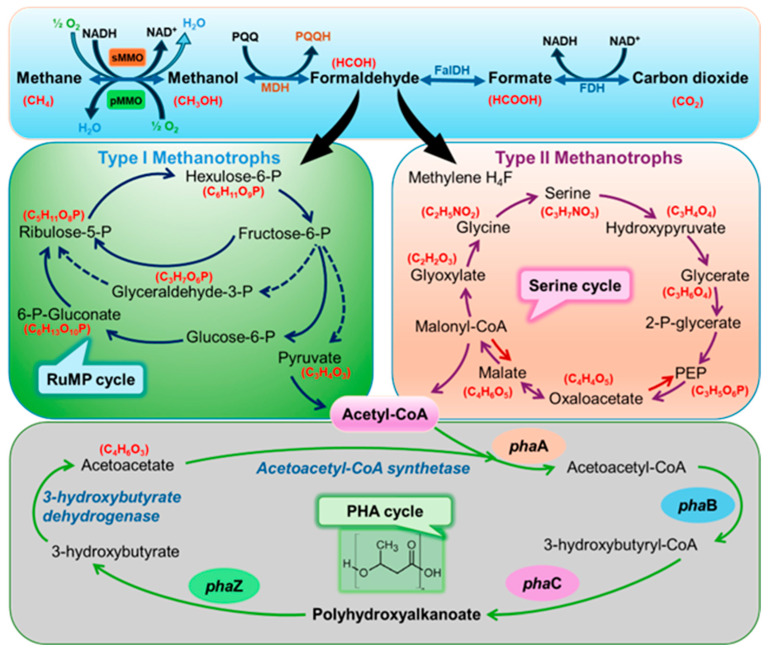
The polyhydroxyalkanoate (PHA) synthesis pathway in the methanotrophs: sMMOs—soluble methane (CH_4_) monooxygenases, pMMOs—particulate CH_4_ monooxygenases, NADH/NAD^+^—nicotinamide adenine dinucleotide, MDH—methanol dehydrogenase, PQQ/PQQH_2_—pyrroloquinoline quinone, FalDH—formaldehyde dehydrogenase, FDH—formate dehydrogenase, RuMP—ribulose monophosphate pathway, PEP—phosphoenolpyruvate, *phaA*—3-ketothiolase, *phaB*—acetoacetyl-CoA-reductase, *phaC*—PHAs synthetase, and *phaZ*—polyhydroxybutyrate depolymerase.

Methanotrophs play a vital role in the global C cycle as they oxidize CH_4_ and potent GHGs to CO_2_ [32,33]. The two main types of methanotrophs are type I and type II, which differ in their physiological and metabolic properties. Type I methanotrophs, including *Methylomonas*, *Methylobacter*, and *Methylomicrobium*, are typically found in environments with high CH_4_ concentrations, such as landfills, rice paddies, and the rumens of ruminant animals [18,34,35]. These microbes are characterized by their potential to rapidly grow and reproduce, making them attractive candidates for biofuel and biopolymer production. In contrast, type II methanotrophs, such as *Methylocystis* and *Methylosinus*, are generally found in environments with lower CH_4_ concentrations and are better adapted to nutrient-limited conditions [1,6]. The metabolism of methanotrophs is characterized by a complex network of enzymatic reactions and pathways that enable them to convert CH_4_ into energy and essential cellular components efficiently. This process typically begins with CH_4_ oxidation to methanol, which is further oxidized to formaldehyde [18,36]. Methanotrophs possess specialized enzymes, such as CH_4_ monooxygenases (MMOs), particulate MMOs (pMMOs), and soluble MMOs (sMMOs), which catalyze the initial oxidation step. The produced formaldehyde is either assimilated into biomass via the ribulose monophosphate (RuMP)/serine pathway or oxidized to CO_2_ for energy production [6,7]. A key aspect of the metabolism of methanotrophs is the interspecies electron transfer that occurs during the anaerobic decomposition of organic matter. Methanotrophs are majorly classified into a subdivision of α-proteobacteria/γ-proteobacteria and exhibit a range of adaptations that allow them to thrive in diverse environments, including acidic conditions [1,13]. A diverse thermoacidophilic group of methanotrophs that is involved in CO_2_ fixation belongs to *Verrucomicrobia*, such as *Acidimethylosilex*, and *Methyloacidiphilium* [6,7]. Type II methanotrophs (α-Proteobacteria) produce PHAs via the serine pathway involving 3-ketothiolase (encoded by *phaA*), acetoacetyl-CoA-reductase (encoded by *phaB*), and PHA synthetase (encoded by *phaC*). In contrast, type I methanotrophs do not exhibit PHA accumulation [37,38,39]. The CH_4_-based PHA production by methanotrophs depends on the culture type (pure methanotrophs or mixed cultures), process parameters, such as CH_4_ concentration, O_2_ availability, age of inoculum, pH value, temperature, presence of copolymer precursors (propionate, valerate, or others), and the extent of nutrient limitations [1,8,37]. Alterations in the metabolism of methanotrophs and co-feeding with desirable copolymer precursors can be beneficial for synthesizing various types of PHAs with suitable properties for broad biotechnological applications [6,28,40]. The details of PHA synthesis by methanotrophs are presented in Figure 1.

## 3. Production of Polyhydroxyalkanoates by Methanotrophs from Methane and Their Physical Characteristics

CH_4_-based PHA production is a promising approach to mitigate CH_4_ emissions and replace petroleum-derived synthetic plastics [27,41]. The process parameters strongly influence PHA production by methanotrophs, especially type II, including nutrient limitations, the ratio of CH_4_ and O_2_, co-substrates (propionate and valerate), and the incubation temperature and period [31]. CH_4_-rich biogas is an accessible feedstock for PHAs by methanotrophs. The production of PHAs by *Methylocystis*-dominated mixed inoculums was evaluated after incubation for 175 d under nonsterile conditions using ammonium (NH_4_^+^; N-source) and CH_4_ [42]. A maximum accumulation of PHAs up to 40% of the cdw by the enriched methanotrophs was noted. Furthermore, supplementation with low (100 mg/L) and high (400 mg/L) valerate resulted in up to 21 and 40% (3-hydroxyvalerate—3HV fraction, mol%) PHA contents of 45 and 30% of the cdw, respectively. The Mw analysis suggested that higher 40 mol% 3HV resulted in lower Mw of 0.93 × 10^6^ than the control (1.20 × 10^6^) and 20 mol% 3HV (1.15 × 10^6^). In contrast, the Mw distribution (polydispersity index, PDI) was higher, up to 2.14, for 40 mol% 3HV compared to the control (1.76). The addition of reducing equivalents, such as formate, exhibited up to a 21% decreased CH_4_ requirement in the case of the valerate supplement system compared to the control (14%) [42]. Although CH_4_ is an inexpensive feedstock, the methanotrophic production of PHAs is limited by slow growth and low CH_4_ solubility. The enhancement of CH_4_ mass transfer can be improved by increasing the agitation, which requires energy inputs and incurs high operational expenses. The use of water-in-oil emulsions can support the superior growth of methanotrophs, and subsequently, PHA production [43]. *Methylocystis parvus* OBBPs showed maximum growth rates of 0.009 and 0.052 h^−1^ under non-shaking and shaking conditions (150 rpm), respectively. Based on Nile red staining, the cells were grown in droplets, and the produced PHAs amounted to 32% of the cdw compared to no PHA production under control conditions. Overall, *M. parvus* OBBP-led PHA production was 67-fold better P(3HB) in the emulsions than in the control [43]. A type II methanotroph, *M. parvus* OBBP, grown with CH_4_ as the sole feed, produced P(3HB) only under nutrient-limiting conditions [44]. Using various co-substrates (100 mg/L), such as 3-hydroxybutyrate, propionate, and valerate, *M. parvus* OBBP produced maximum PHAs contents of 60, 32, and 54% of the cdw compared to the control, with 50% of the cdw (as CH_4_ feed) after 48 h of incubation. Among these co-substrates, propionate and valerate exhibited 8 and 22 mol% 3HV in the produced PHAs. Furthermore, increasing the valerate concentration to 2000 mg/L resulted in a higher 40 mol% for 3HV in the accumulated PHAs. Under similar conditions, another type II methanotroph, *Methylosinus trichosporium* OB3b, using CH_4_ and valerate (100 mg/L), produced PHA contents (50% of the cdw) with 20 mol% of 3HV. The produced PHAs using CH_4_ only and with valerate co-substrate as 22 and 37 mol% of 3HV showed melting temperatures of 178, 150, and 134 °C with glass transition temperature (T_g_) values of 8 (typical for P(3HB)), −2, and −6 °C (typical for P(3HB-co-3HV)), respectively. Furthermore, the produced P(3HB), P(3HB-co-3HV) with 22 mol% 3HV, and P(3HB-co-3HV) with 37 mol% 3HV exhibited corresponding Mw values of 3.24 × 10^6^, 1.83 × 10^6^, and 1.70 × 10^6^, with PDI values of 1.67, 1.88, and 2.23, respectively [44]. Under N limitations for incubation up to 310 d, methanotrophic communities were enriched using different CH_4_ concentrations of 20.0, 2.0, and 0.2 g of CH_4_/m^3^ in stirred tank reactors (STRs). R1, R2, and R3 were evaluated for PHA production, respectively [45]. Proteobacteria dominated the microbial population, and the CH_4_-oxidizing culture belonged to type I methanotrophs; *Methylobacter*, *Methylosarcina*, *Methylosoma*, and *Methylomicrobium* were especially abundant in the R1 and R2 STRs. Their populations gradually declined over the incubation period under fed-batch cultivation. *Methylocystis* (type II methanotrophs) were present in these STRs, and their abundance gradually increased in the R2 and R3 STRs. Here, the higher abundance of type I than type II methanotrophs may be associated with high copper content. The methanotroph PHA content (*w*/*w*) in these STRs varied from 0.3 to 0.5% in R1, from 2.9 to 9.7% in R2, and from 0.1 to 0.8% in R3. The composition of PHAs in P(3HV):P(3HB) was 12:1 in R1 and 4:1 in R3. In contrast, a low P(3HV):P(3HB) ratio of 1:7 was observed for R2. The maximum PHA production of 12.6% was recorded in R2, which was cultivated at a higher CH_4_/biomass ratio under N limitation conditions. These differences in the PHA contents in the STRs may be associated with diverse methanotrophic communities [45]. In another study, the highest PHA contents of 1.0, 12.6, and 1.0% of the cdw by enriched inoculums of methanotrophs was supported by corresponding feedings of 20, 2.0, and 0.2 g of CH_4_/m^3^, respectively [46]. Under P-limiting conditions, *Methylocystis* sp. GB25 showed production of PHAs of 46.2% with a productivity of 1.13 g/L·h and Mw of 2.41 × 10^6^ [47]. Owing to the limitations of K, S, and Fe, the *Methylocystis* sp. GB 25 DSM 7674-dominated mixed culture resulted in PHA accumulations of 33.6, 32.6, and 10.4% of the cdw, respectively [48]. Under P deficiency, the synthesized PHA achieved an Mw of 3.1 × 10^6^.

*Methylocystis* sp. WRRC can produce co-polymers P(3HB-co-3HV) of PHAs by co-feeding CH_4_ with valerate or pentanol [49]. Using pure CH_4_, a maximum PHA production of 0.20 g/L was observed with 0.38 g of PHA/g of CH_4_. The co-feeding of pentanol and valerate exhibited higher PHA production rates of 0.31 and 0.57 g/L, with PHA contents up to 78% of the cdw, respectively. The synthesized P(3HB), P(3HB-co-15%-3HV), and P(3HB-co-43%-3HV) showed melting temperatures of 180, 161, and 170 °C with % crystallinity values of 52, 23, and 5%, respectively. These findings suggest that the PHA yield and polymeric characteristics can be altered by cofeeding valerate and CH_4_ [49]. García-Pérez et al. [50] evaluated the influence of nutrient-limiting environments, such as N, K, and Mn, with a surplus of Fe on P(3HB) production by *Methylocystis hirsuta* DSMZ 18500. The accumulation of PHAs was higher at 28% of the cdw under these nutrient-limiting conditions than in the control (7.5% of the cdw). Overall, the maximum CH_4_ elimination capacity and PHA content and productivity were recorded as 16.2 g/m^3^·h, 34.6% of the cdw, and 1.4 kg/m^3^·d [50]. Cantera et al. [51] assessed the feasibility of various value-added products using CH_4_, including PHAs, by mixing cultures in bubble-column bioreactors with different magnesium concentrations (Mg^2+^; 0.2, 0.02, and 0.002 g/L). High concentrations of Mg^2+^ were effective in producing ectoine (94.2 mg/g of the cdw) and EPS (2.6 g/L). In contrast, low Mg^2+^ favored PHA production of 14.3 mg/L. The dominant organisms in the reactors are *Halomonas*, *Marinobacter*, *Methylophaga*, and *Methylomicrobium* [51]. Similarly, Wendlandt et al. [52] reported PHA production rates of 28.3–51.3% of the cdw by *Methylocystys* sp. GB25 under Mg^2+^-limiting conditions. In another study, *M. trichosporium* OB3b and *M. parvus* OBBP under N- and Mg^2+^-limiting conditions exhibited higher PHA accumulation rates of 29–60% of the cdw [53].

The prices of selected biodegradable plastics are approximately USD 2.60–5.80/kg for starch-based bioplastics, USD 2.00–3.45/kg for poly(lactic acid), and USD 2.00–6.50/kg for P(3HB) [54]. The pilot production design and economic evaluation for 100,000 t/a of P(3HB) by methanotrophs and utilizing extraction by acetone/water solvent methods suggested that the estimated PHA expenses varied in ranges from USD 4.1/kg to USD 6.8/kg [54]. The production of PHAs by thermophilic methanotrophs operating at 60 °C can be beneficial over mesophilic methanotrophs (30–45 °C) by reducing costs to USD 3.2–5.4/kg. High-temperature fermentation decreases the mass transfer of CH_4_. A few thermophilic methanotrophs can accumulate PHAs. Additionally, thermophilic PHA production has several advantages, such as higher substrate solubility, increased diffusion rates, and low contamination risks [54]. Alternatively, biogas feed as a source of CH_4_ to produce PHAs by *M. hirsuta* could be helpful in developing anaerobic digestion biorefineries [55]. The artificially designed biogas feed (CH_4_, 70%; CO_2_, 29.5%; and H_2_S, 0.5%) showed a PHA production rate of 45% of the cdw by *M. hirsuta* DSM 18500. Here, H_2_S did not negatively influence *M. hirsuta* DSM 18,500 growth on the additional supplementation of volatile fatty acids, and a maximum PHA content and yield were recorded up to 54% of the cdw and 0.63 g of PHAs/g of substrate. Further, valerate supplementation resulted in 13.5 mol% of 3HV [55]. The enrichment temperature influences PHA production by mixed methanotroph inoculums. The mixed culture enrichment at temperatures of 30 and 37 °C resulted in PHA production rates of up to 35.1% and 34.1% of the cdw, respectively [56]. Here, high PHA production in these methanotroph-mixed inocula was associated with the dominance of *Methylocystis* at ~30% of the total microbial population. In another study, a *Methylosinus*-dominated mixed culture from CH_4_ showed a low PHA production rate of 8.6% of the cdw as P(3HB) [57]. Furthermore, supplementing propionate and valerate as co-substrates with CH_4_ enhanced it to 22.6 and 65.0 mol%, respectively, with a maximum PHA production rate of 14.1% of the cdw.

The enriched methanotrophic cultures from diverse inocula, *Sphagnum* peat moss, and *Sphagnum* with activated sludge, dominated by *Proteobacteria* (35.7–62.1%) and *Bacteroidetes* (31.7–39.8%), exhibited PHA production of up to 13.6% of the cdw [58]. In contrast, thermophilic conditions mixed with CH_4_-utilizing culture showed PHA production of up to 10.0 and 8.0% of the cdw, with 3HV contents of up to 70 and 85 mol% at 55 and 58 °C, respectively [59]. Eam et al. [60] examined bioaugmentation of P(3HB)-producing methanotrophs in activated sludge using *M. trichosporium* OB3b to improve PHA production. The 1:1 ratio of activated sludge to *M. trichosporium* OB3b exhibited the maximum PHA production of 37.1% of the cdw. In *M. trichosporium* OB3b-amended cultures, measurements of the microbial dynamics revealed that the dominant type II methanotrophs *Methylocystis* and *Methylophilus* spp. were crucial for the production of PHAs [60]. The co-feeding of CH_4_ and CO_2_ positively influenced PHA production by type II methanotrophs and enhanced PHA production by up to 162% [61]. The maximum reported PHAs produced was 38.0% of the cdw by *Methylocystis* sp. MJC1. Metabolic flux analysis revealed that 45% of the total PHA production occurred from CO_2_ through the vital roles of phosphoenolpyruvate carboxylase and crotonyl-CoA carboxylase/reductase [61]. Genomic analysis of the thermophilic methanotrophs *Methylocaldum* spp. metabolism (*M. szegediense* (O-12 and Norfolk) and *M. marinum* S8) revealed them as PHA producers [62]. In addition, *M. parvus* OBBP produced PHAs up to 35% of the cdw from ethane as the sole C source, compared to 48.0% of the cdw using CH_4_ as a feed [63]. Furthermore, co-feeding with ethane and valerate resulted in 25 mol% of the 3HV contents in the total PHA production of 12.9% of the cdw. Nitrate proved to be a better N source to produce PHAs by up to 27% of the cdw in mixed methanotrophic cultures dominated by *Methylocystis* sp. than the NH_4_^+^ [64]. The PHA-producing mixed cultures enriched from landfill biocover soil, peat bog soil, and waste-activated sludge produced PHA copolymers with diverse ratios of CH_4_ and O_2_. These cultures showed a maximum PHA production of up to 41 mol% 3HV after supplementing with valerate. The waste-activated sludge-based culture exhibited the highest PHA yield of 0.42 g/g of substrate at 10% of CH_4_ feed [64]. Similarly, the 0.24–0.34 g yield of PHB/g of CH_4_ with a concentration and contents of 0.14–0.28 mg/mL and 30.5–50.3% of the cdw by *M. parvus* OBBP was reported [65]. In the methanotrophic metabolism for PHA production from CH_4_, diverse C assimilation pathways and reduced power requirements influence the final storage of PHAs. In addition, formate supplementation of P(3HB)-rich cells delayed PHB consumption. In a previous study, a mixed culture (*M. parvus* OBBP and *Methylosinus* sp. LW4, *Methylocystis* 42/22, and *Methylocystis* KS30) exhibited PHA production of 25% of the cdw [66]. Potential methanotrophs types I (*Methylocaldum* O11a, *Methylomicrobium album* BG8, and *Methylomonas* LW13) and II (*Methylocapsa* acidiphila, *Methylocystis* 42/22, *M. hirsuta* CSC1, *M. parvus* OBBP, *Methylocystis* strain M, *Methylocystis rosea* SV99, *Methylosinus* sp. LW4, *Methylocystis* SC2, *M. trichosporium* OB3b, and *Methylosinus sporium*) were evaluated by Pieja et al. [67]. Among these cultures, the type II methanotrophs exhibited PHA production rates of 7.0–36% of the cdw, which was evident with *phaC*. In addition, the methanotrophic-enriched cultures showed a PHA production rate of up to 46% of the cdw [67].

Various extraction procedures, including solvent extraction (1,3-dioxolane), cell lysis followed by solvent extraction, and cell lysis without solvents, have been evaluated for efficient recovery of intracellular PHAs [68]. Among these methods, the integrative process of cell lysis followed by solvent extraction proved beneficial for achieving 91% PHA recovery with 93% purity. *Methylocystis* sp. GB 25 synthesized PHAs from CH_4_ and CO_2_ with a Mw of 1.08 × 10^6^ and a melting temperature of 175 °C [69]. The biodegradable PHAs produced by the type II methanotroph *Methylocystis* sp. MJC1 exhibited a high biomass of 21.3 g cdw/L through a high-cell density cultivation approach [70]. The maximum PHAs produced was reported as 8.9 g/L (41.9% of the cdw) with 28 mol% 3HV. Waste gases and C sources, especially C1, such as CH_4_, CO_2,_ and methanol, originating from anthropogenic and industrial activities, can be directly utilized to produce value-added biomolecules, such as PHAs, at up to 88% of the cdw by methanotrophs [41]. Biomass yields, PHA production, and microbial populations under high-resource (CH_4_, 20% and NH_4_^+^, 10 mM) or low-resource (CH_4_, 0.2% and NH_4_^+^, 0.1 mM) conditions influence PHA production by *Methylocystis* [39]. PHA production by *Methylocystis* was observed under high-resource conditions, with a yield of 12.6% cdw. In the case of low resources, the hindrance of PHA production by mixed inocula is associated with a lack of dominant methanotrophs [39]. Simulation analysis of PHA production suggested that *M. hirsuta* can produce up to 51.6% of the cdw in a forced-liquid vertical loop bioreactor [71]. In another simulation study, methanotrophs could efficiently produce PHAs with a yield of 0.32 kg/m^3^/d with a low valerate concentration using CH_4_-abundant gas streams [29]. Process simulation and design, such as superficial gas velocity, aspect ratio, reactor volume, and diameter, positively influenced PHA production by methanotrophs [72]. PHA production using CH_4_ in bubble column bioreactors showed an accumulation of 37.5% of the cdw by *Methylocystis* after 12 d. In a 14 L fermenter for PHA productions under nitrate-limiting conditions, the mixed methanotrophic culture dominated by *Methylocystis* accumulated PHAs up to 22.2% of the cdw. Analysis of the physical characteristics suggested that synthesized PHAs had a Mw 2.2 × 10^5^ g/mol with a PDI of 1.82 [73]. The PHAs produced were up to 3.0 and 7.4% of the cdw by the methanotrophic bacterium strain MTS using CH_4_ and methanol as feed, respectively [74]. Furthermore, intracellular PHA degradation in type II methanotrophs was confirmed using ^13^C nuclear magnetic resonance under anoxic conditions. Wendlandt et al. [75] evaluated PHAs produced by *Methylocystis* sp. GB of 25 DSM7674 under non-sterile conditions. Under the continuous mode and nutrient phosphate limitation, PHA contents of up to 51% of the cdw were produced, with a high Mw of 2.5 × 10^6^. Xin et al. [76] determined the Mw of PHAs produced by *M. trichosporium* IMV 3011, *M. trichosporium* OB3b, *Methylococcus capsulatus* HD6T, and *Methylomonas* sp. GYJ3 under different conditions, and substrates such as CH_4_ and methanol were variable in the range of 0.30–1.4 × 10^6^. The Mw of PHAs is highly dependent on the type of microorganism and the critical enzyme activity needed for PHA metabolism. In another study, the co-feeding of CH_4_ and methanol resulted in PHA accumulation of up to 40% of the cdw, with a high Mw of 1.28–1.48 × 10^6^ by *M. trichosporium* IMV3011 [77]. High methanol production by PHAs containing *M. trichosporium* IMV3011 cells was observed [37]. After 168 h of incubation, a maximum PHA production of 41.0% of the cdw was observed in a mineral salt medium containing copper (2 mg/L). A two-phase partitioning bioreactor and silicon oil-based cultivation of *Methylobacterium organophilum* CZ-2 showed PHA production of 38.0% of the cdw, compared to mixed consortium (34% of the cdw with productivity of 1.61 mg of PHAs/g·h) [78]. Co-feeding with citrate or propionate resulted in PHA production of 5.0–617 mg/L by *M. organophilum* CZ-2 [79].

Malvar et al. [80] developed a rheological method to identify PHA-producing bacteria. Under N depletion conditions, methanotrophs consortia showed PHA production up to 0.19 g/L under incubation for 120 h. This finding suggests a correlation between bacterial motion and PHA production. A mechanistic model was evaluated to enhance PHA production in *M. hirsuta* using biogas as a feed under nutrient-limiting conditions [81]. The biomass growth and PHA production on CH_4_ feed (0.14 and 0.25 g of chemical oxygen demand (COD)/g of COD), a CH_4_ affinity constant of 5.1 g of COD/m^3^), and an O_2_ affinity constant of 4.1 g of O_2_/m^3^ showed a PHA production rate of 0.39 g of COD/g of COD/d on CH_4_. In another study, *M. hirsuta* CSC1 genome sequence analysis suggested that it could be a potential PHA producer [35]. Methanotrophs enhance biomass growth and survival under variable and nutrient-limiting conditions owing to their metabolic flexibility. The H_2_ metabolism in *Methylacidiphilum* sp. RTK17.1 Verrucomicrobial methanotrophs is principally linked to energy management, and its consumption offers a competitive growth advantage within hypoxic habitats [82]. Myung et al. [83] established the synthesis of various PHA co-polymers by *M. parvus* OBBP with supplementation of the corresponding co-substrates, such as poly(3-hydroxybutyrate-co-4-hydroxybutyrate) (P(3HB-co-4HB)), poly(3-hydroxybutyrate-co-5-hydroxyvalerate-co-3-hydroxyvalerate) (P(3HB-co-5HV-co-3HV)), and poly(3-hydroxybutyrate-co-6-hydroxyhexanoate-co-4-hydroxybutyrate) (P(3HB-co-6HHx-co-4HB)) using butyrate or 4-hydroxybutyrate (4HB), valerate or 5-hydroxyvalerate (5HV), and hexanoate or 6-hydroxyhexanoate (6HHx), respectively. The maximum PHAs produced were up to 59% of the cdw, with corresponding 3HV (25 mol%), 4HB (9.5 mol%), 5HV (3.6 mol%), and 6HHx (1.4 mol%). These co-polymers’ Mw, melting temperature, and T_g_ varied in ranges of 1.22 × 10^6^–1.33 × 10^6^, 135–150 °C, and −1–−5 °C, respectively [83].

The cost-effective production of PHAs by methanotrophs to manufacture biodegradable plastics was examined. Based on an analysis of CH_4_ emissions from Australian landfills, the outcomes suggest that economically viable bioplastic production is feasible considering the competitive price of synthetic plastics. The waste of small (5000 t/y), medium (35,000 t/y), and large (230,000 t/y) landfills can recover 162–7480 t of CH_4_ to produce 71–3252 t of PHAs, with a cost of 1.5–2.0 AUD/kg of PHAs [84]. The PHAs (P(3HB-co-3HV)) were synthesized using CH_4_ and alkenoates (produced from recycled PHAs via catalytic pyrolytic depolymerization) by *M. parvus* OBBP with a Mw in the range of 1.18–1.47 × 10^6^ and a purified yield of 33–47 mg of PHAs [85]. The potential of type I and type II methanotrophs to convert biogas containing H_2_S into PHAs was validated by transcriptomic analysis. Metabolic analysis suggested that the native pathway of H_2_S utilization involves type II methanotrophs. Under fed-batch conditions, the *Methylocystis* sp. MJC1 and *Methylocystis* sp. OK1 showed biomass growth of 4.0, and 4.5 g cdw/L, respectively. *Methylocystis* sp. showed a maximum PHA production rate of 2.9 g/L using biogas [86]. The pH range of 5.5 to 7.0 highly supports the growth of *Methylocystis*. A maximum PHA production rate of 43.7% of the cdw was reported at pH 5.5 in mixed methanotrophic cultures dominated by *Methylocystis* under N-deprived conditions [87]. At pH 8.5, the abundance of *Methylocystis* declined from 14% to 85–90% at a pH of 5.5–7.0.

*Methylocystis* sp. MJC1 cultured in 3:7 CH_4_ and air (*v*/*v*) resulted in biomass growth and PHA production of 52.9 and 28.4 g/L at an incubation time of 120 h, respectively [88]. A low PHA production (0.20 g/L·h) was reported because of the long incubation period. Interestingly, increasing the O_2_-to-CH_4_ supply to a ratio of 1.5 showed a 1.5-fold increase in biomass productivity and delayed PHA production. At an equal ratio of CH_4_ and O_2_, the maximum biomass achieved was 55.9 g/L, with PHA production of 34.5 g/L (61.7%) showing a productivity of 0.36 g/L·h after 164 h of incubation [88]. *Methylomonas* sp. DH-1 and *M. trichosporium* OB3b co-cultures have the potential to produce PHAs from CH_4_ and CO_2_ under C-rich or C-lean conditions without an external supply of O_2_ [32]. Using simulated biogas, the syntrophic association of these methanotrophs resulted in biomass and PHA production of 1130 and 83.0 mg/L, respectively, in the presence of a 2.9 mM N source. Under N-limiting conditions, *Methylocystis* spp. MJC1 produced PHAs up to 44.5% of the cdw [89]. Genomic investigations have suggested that *Methylocystis* sp. MJC1 contains pMMO and sMMO particulates that are not commonly reported in *Methylocystis* spp.

A numerical simulation analysis was conducted to describe the growth and PHA production by methanotrophs based on the roles of the gas flow rate, metabolic heat release, reactor pressure, and pH [90]. This analysis suggested that a high mass-transfer rate and high-pressure operational conditions are undesirable for achieving high PHA productivity. For the first time, Chau et al. [40], confirmed PHA production by the type I methanotroph *Methylotuvimicrobium alcaliphilum* 20Z. It exhibited production of 3HB and P(3HB) of 334 mg/L and 1.3% of the cdw upon co-feeding with CH_4_ and xylose. *Methylosinus*-dominant (54%) consortia produced PHAs of 183 mg/L from 12.3% (*v*/*v*) of CH_4_ and Cu (10 µM) in the media [91]. Remarkably, this consortium tolerated a high CH_4_ content of up to 70% (*v*/*v*). PHB, as an intracellular reducing agent, helps produce high levels of methanol 400 mg/L by *M. hirsuta* via a 75% reduction in formate requirements [92]. *M. hirsuta* CSC1 exhibited better PHA production at room temperature over 15 and 37 °C. A maximum PHA content of 45% of the cdw was reported at a 2:1 ratio of O_2_ to CH_4_ [93]. Under N-limiting conditions, *M. parvus*, a type II methanotroph, can produce PHAs up to 50% of the cdw [94]. The AD sludge-based enriched CH_4_-utilizing mixed cultures produced P(3HB) of approximately 51.0% of the cdw from CH_4_ [95]. Furthermore, supplementation with valerate enhanced the PHAs up to 52.0%, with a 3HV content of 33 mol%. Response surface methodology was used to optimize PHA production by *M. trichosporium* OB3b by co-feeding with methanol and CH_4_ [96]. The maximum PHAs produced were reported to be up to 48.7 mg/L with a cdw content of 52.5%. The methanotroph population enriched from compost soils and landfill top cover showed PHA production of up to 25 mg/g of the cdw using 40% CH_4_ as feed [97]. Details of PHA production by methanotrophs are presented in Table 1.

## 4. Genetic Engineering in Methanotrophs for Producing Polyhydroxyalkanoates (PHAs)

Polyhydroxyalkanoates (PHAs) are biopolymers with enormous potential for numerous applications, including biodegradable plastics. Methanotrophs exploit CH_4_ as their carbon and energy source and are promising producers of PHAs [6,94]. One key advantage of using methanotrophs for producing PHAs is the availability of CH_4_ feedstock as a GHG source. Methane (CH_4_) is the chief constituent of natural gas and can be obtained through the AD of organic wastes of agricultural, municipal, industrial, or synthetic origins. Methanotrophs convert CH_4_ into PHAs, which can then be extracted and processed in a manner similar to traditional petroleum-derived plastics [1,8]. Current research is focused on expanding the range of PHAs synthesized by methanotrophic bacteria [6,40]. Although early investigations have demonstrated the production of PHAs, their ability to produce other PHAs has been limited because P(3HB) is a major product [6,67]. Genetic engineering approaches have shown promise in broadening the monomer composition of PHAs formed by methanotrophs. Researchers have demonstrated PHA production with varying chain lengths and functional groups by manipulating the metabolic pathways and enzymatic activities within methanotrophic bacteria [18,37,83]. This allows for tailoring the material properties of biopolymers, such as their mechanical strength, flexibility, and biodegradability, to suit specific biotechnological applications. For the first time, Bordel et al. [98] demonstrated a type II methanotroph genome-scale metabolic model construction in *Methylocystis hirsuta.* Directing type II methanotrophs through metabolic engineering for high C fluxes via acetoacetyl-CoA under N-limited conditions could be a valuable platform for producing PHAs [23,98]. Engineered *M. trichosporium* OB3b through reconstructing 4HB biosynthetic pathways, such as NADPH-dependent succinate semialdehyde-reductase and CoA-dependent succinate semialdehyde-dehydrogenase, showed 3.08 mol% of 4HB in the produced PHAs (P(3HB-co-4HB)) from CH_4_ [99]. The *Bacillus subtillis* 168 lipase A gene was successfully expressed in *M. trichosporium* Ob3b to produce lipase and PHAs simultaneously [100]). The engineered methanotrophs exhibited maximum PHA production rates of 191 mg/L and 71.5 U/mg of lipase activity. In vivo quantification of PHAs by rapid and efficient approaches in a *Methylocystis* sp. A Rockwell model was demonstrated by Lazic et al. [101]. In contrast, PHA polymerase (*phaC*) deletion in *M. trichosporium* OB3b showed a high production of 2-hydroxyisobutyric acid (30.0 mg/L) and 1,3-butanediol (60.5 mg/L) from CH_4_ as feed [38]). In addition, few simulation studies have been demonstrated producing P(3HB) by methanotrophs using natural gas [102,103]. Overall, the use of genetically engineered methanotrophic bacteria for PHA production represents a promising approach to address the challenges associated with environmental pollution and traditional petroleum-based plastics.

## 5. Perspectives and Conclusions

The production of PHAs is an emerging alternative to conventional petroleum-derived synthetic plastics owing to its biodegradability and sustainability. These biopolymers can be formed using various renewable sources, including agricultural waste, which can help diminish the environmental pollution caused by plastic waste. Methanotrophs have garnered considerable attention because of their broad applications in numerous areas, such as bioremediation, biotechnology, and biofuel production. One particularly promising aspect of methanotrophs is their ability to synthesize PHAs, which are a class of biodegradable and renewable bioplastics. Methanotrophs produce PHAs under certain growth conditions. However, the commercial-scale production of PHAs by methanotrophs faces several challenges and limitations. A key challenge is the low yield and productivity of PHAs from methanotrophs compared to other microbial sources. To address these challenges, researchers have explored alternative substrates and cultivation strategies for PHA production using methanotrophs. For instance, using agricultural waste-derived biogas as a source of CH_4_ feedstock could offer a more sustainable and lucrative approach while reducing the environmental impact of waste. Therefore, integrating PHA production with other bioprocesses, such as wastewater treatment and biogas generation, could enhance the overall sustainability and economic viability of the process. PHAs have been extensively investigated for biomedical applications, such as implants and drug delivery systems, owing to their inherent biocompatibility and biodegradability. The use of additive manufacturing techniques, such as 3D printing, has further expanded the potential applications of PHB in the biomedical field, allowing for the fabrication of customized complex structures. In addition, PHA properties (physical and chemical), such as their mechanical strength, biodegradability, and biocompatibility, can be tailored by copolymerizing different hydroxyalkanoate monomers. This versatility makes PHAs produced by methanotrophs valuable materials for biomedical applications, such as tissue engineering, drug delivery, and medical implants. Recent legislation, such as the Circular Economy Action Plan 2020, has driven the development of more sustainable production processes and better end-of-life solutions for bio-derived plastics. By addressing the challenges and limitations of PHA production by methanotrophs, researchers can contribute to the development of a more circular and sustainable plastics economy. Further improvements in fermentation technology, metabolic engineering, and process optimization are required to increase the production efficiency of PHAs. In conclusion, the production of PHAs by methanotrophic bacteria represents a promising approach for addressing environmental concerns associated with traditional plastic production. However, further research and development is essential to overcome the technological and ecological challenges that currently limit the large-scale adoption of this technology.

## Figures and Tables

**Table 1:** Polyhydroxyalkanoate (PHA) production potentials of methanotrophs from methane (CH_4_).

Methanotrophs	CH_4_ (%)	Fermentation Conditions (Mode/Working Capacity (L)//Incubation Period (h))	PHAs		Reference
			% in cdw	Mw (×10^6^)	
Enriched methanotrophs/consortia	25	Batch/70.0/24	46.2	2.41	[47]
	25	Batch/70.0/24	10.4–33.6	1.81–3.10	[48]
	50	Batch/0.05/24	46.0	-	[67]
	80 ^a^	Continuous/2.00/384	34.0	-	[78]
	150 ^b^	Continuous/4.00/24	25.0	-	[66]
	5	Continuous/0.40/432	12.6	-	[45]
	5	Continuous/0.40/310	1.0–12.6	-	[46]
	50	Batch/0.05/48	39.0–45.0	0.93–1.20	[42]
	40	Batch/0.20/480	25.0 ^d^	-	[97]
	46 ^a^	Continuous/2.00/150	0.01 ^e^	-	[51]
	50	Batch/0.05/48	51.0	-	[95]
	50	Semi-continuous/0.24/72	8.6–14.1	-	[59]
	50	Batch/0.03/48	8.0–10.0	-	[57]
	177 ^b^	Batch/0.20/-	34.1–35.1	-	[56]
	161 ^b^	Batch/0.20/384	13.6	-	[58]
	- ^c^	Batch/0.05/192	0.19 ^e^	-	[80]
	12.3	Batch/-/268	0.18 ^e^	-	[91]
	50	Continuous/8.00/120	22.2	2.20	[72]
	20	Batch/0.05/144	12.6	-	[39]
	30	Batch/0.04/168	12.9	-	[64]
	9	Batch/2.50/192	43.7	-	[87]
Activated sludge and *Methylosinus trichosporium* OB3b	50	Batch/0.04/72	37.1	-	[60]
*Methanotrophic bacterium* MTS	25	Batch/0.30/-	3.00	-	[74]
*Methylobacterium organophilus* CZ-2	80 ^a^	Continuous/2.00/384	38.0–39.0	-	[78]
	42 ^a^	Continuous/2.00/240	88.0	-	[79]
*Methylococcus capsulatus* HD6T	50	Batch/0.10/120	-	0.95	[76]
*Methylocystis*	4	Semi-continuous/400/-	37.5	-	[72]
*Methylocystis* 42/22	50	Batch/0.05/24	25.0	-	[67]
*Methylocystis* SC2	50	Batch/0.05/24	30.0	-	[67]
*Methylocystis hirsuta* CSC1	50	Batch/0.05/24	7.0	-	[67]
	29.2	Batch/0.40/504	45.0	-	[93]
*M. hirsuta* DSMZ 18500	4	Batch/2.50/1632	28–34.6	-	[50]
	35	Batch/0.05/168	45.0–54.0	-	[55]
*M. hirsuta*	50	Continuous/10.0/120	51.6	-	[71]
*Methylocystis parvus* OBBP	50	Batch/0.05/66	30.5–50.3	-	[65]
	50	Batch/0.05/24	36.0	-	[67]
	30	Batch/0.05/22	60.0	-	[53]
	40	Batch/0.05/24	48.0–64.0 ^d^	1.18–1.47	[85]
	40	Batch/-/168	32.0	-	[43]
	40	Batch/0.05/48	32.0–60.0	-	[44]
	40	Batch/0.05/48	59.0	1.22–1.33	[83]
	40	Batch/0.05/24	35.0–48.0	-	[63]
*M. parvus*	74 ^b^	Batch/0.02/-	50.0	-	[94]
*Methylocystis rosea* SV99	50	Batch/0.05/24	9.00	-	[67]
*Methylocystis* sp. MJC1	30	Batch/0.05/96	41.9	-	[70]
	30	Batch/3.00/96	44.5	-	[89]
	30	Batch/2.50/208	61.7	-	[88]
	30	Batch/1.20/140	2.90 ^e^	-	[86]
	20	Batch/0.10/24	38.0	-	[61]
*Methylocystis* sp. GB25	20	Batch/70.0/24	28.3–51.3	-	[52]
	15	Batch/70.0/24	45.0–51.0	2.50	[75]
	-	Batch/30.0/504	-	1.08	[69]
*Methylocystis* strain M	50	Batch/0.05/24	14.0	-	[67]
*Methylocystis* sp. WRRC1	50	Batch/0.02/72	0.20–0.57 ^e^	-	[49]
*Methylomonas* sp. GYJB	50	Batch/0.10/120	-	0.30	[76]
*Methylosinus* sp. LW4	50	Batch/0.05/24	100	-	[67]
*Methylosinus sporium*	50	Batch/0.05/24	9.00	-	[67]
*M. trichosporium* IMV3011	50	Batch/0.10/120	-	1.20	[76]
	50	Batch/0.05/168	41.0		[37]
	50	Batch/0.10/144	38.1	1.50	[77]
*M. trichosporium* OB3b	50	Batch/0.05/24	38.0		[67]
	50	Batch/0.10/120	-	0.95	[76]
	80 ^a^	Continuous/2.00/384	57.0	-	[78]
	30	Batch/0.05/28	29.0–60.0	-	[53]
	50	Batch/0.10/120	52.5	-	[95]
*Methylotuvimicrobium alcaliphilum* 20Z	-	Batch/0.10/168	1.30	-	[40]
*Methylomonas* sp. DH-1 and *M. trichosporium* OB3b	30	Batch/0.10/168	0.08 ^e^	-	[32]

^a^ CH_4_ in mg/L·h, ^b^ CH_4_ in mg/L; ^c^ not available or reported; ^d^ PHAs in mg or mg/g of cdw; ^e^ PHAs in g/L.
